# Exploring syndemic vulnerability among adolescents living in urban cities in the Netherlands: a latent class analysis

**DOI:** 10.1136/bmjph-2024-002032

**Published:** 2026-01-19

**Authors:** Samantha Frederika Francisca Groenestein, Sabine Plag, Eveline Margaretha Dubbeldeman, Robert R J M Vermeiren, Jet Bussemaker, Suzan van der Pas, Matty R Crone

**Affiliations:** 1 Department of Public Health and Primary Care, Leiden University Medical Center/Health Campus The Hague, Leiden, The Netherlands; 2 Leiden University, Leiden, The Netherlands; 3 Department of Child and Adolescent Psychiatry, Leiden University Medical Center, Leiden, The Netherlands; 4 Institute of Public Administration, Leiden University, Leiden, the Netherlands; 5 Faculty of Social Work & Applied Psychology, Leiden University of Applied Sciences, Leiden, The Netherlands; 6 Department of Health Promotion, Maastricht University, Maastricht, The Netherlands

**Keywords:** Adolescent, Comorbidity, Syndemic, Public Health, Cross-Sectional Studies

## Abstract

**Background:**

The increase of adolescent multimorbidity in welfare states is a major concern for current and future population health. However, current health interventions remain insufficient. Syndemic research among adolescents in high-income countries could improve understanding of mechanisms contributing to social and health inequalities.

This study explores the existence of clustered health conditions among adolescents aged 10–19 registered at general practices in two average Dutch cities: The Hague and Leiden. We examine which social and contextual factors are associated with these clustered health conditions and to what extent these clusters relate to healthcare use and costs.

**Methods:**

This cross-sectional study used general practitioner registration data on diagnoses from the Extramural Leiden University Medical Centrum Academic Network database to explore multimorbidity based on the 2-year prevalence of 20 health conditions among adolescents (n=10 841). Latent class analysis was applied to distinguish multimorbidity classes. Statistics Netherlands provided information on factors at the individual (eg, country of origin), family (eg, divorced parents) and household (eg, income) levels. Multinomial logistic regression analysis was applied to identify multimorbidity class differences in these social contextual factors, healthcare use and costs.

**Results:**

In 2018 and 2019, 25.7% (n=2781) of adolescents had at least two health conditions. We identified four classes characterised by a high probability of health conditions: ‘asthma-skin conditions’ (n=442), ‘skin conditions-pain’ (n=794), ‘externalising behavioural disorders and skin conditions’ (n=565) and ‘gastrointestinal conditions, internalising disorders and pain’ (n=980). Adolescents in classes characterised by externalising or internalising conditions combined with physical and somatic health conditions were most often linked to several more prominent adverse social contextual factors, such as early school dropout. Increased healthcare use and costs across all classes indicated a higher disease burden for adolescents with clustered health conditions compared with those with one or no health conditions.

**Conclusions:**

Disease clusters are prevalent in adolescents living in Dutch cities, driven by social contextual factors at the individual, household and parental levels, and show increased healthcare use and costs. Our results support the need for integrated preventative strategies focusing on multi-level aspects.

**WHAT IS ALREADY KNOWN ON THIS TOPIC**

- Multimorbidity affects adolescents’ current and future health, well-being and social participation.

**WHAT THIS STUDY ADDS**

- This study advances our understanding of adolescents’ syndemic vulnerability.
- Adolescents with combined health conditions are more likely to also have parents with physical, somatic or mental health conditions.
- Adolescents combining health conditions with internalising or externalising mental health problems more often show disadvantages in social participation.

**HOW THIS STUDY MIGHT AFFECT RESEARCH, PRACTICE OR POLICY**

- An integrated approach by healthcare providers and social support systems is needed to ensure that diagnostic and treatment strategies adequately account for the interaction and co-occurrence of health and social conditions.

## Introduction

 The ongoing increase in multimorbidity, defined as the co-occurrence of two or more health conditions, is a major concern for current and future population health. While multimorbidity is mostly linked to ageing populations, it also adversely affects younger groups’ health, well-being and social participation. Moreover, some chronic health conditions that emerge during adolescence persist into adulthood, contributing to adverse health outcomes and reinforcing social inequalities later in life.^1 2^

Shared exposure to a set of adverse social conditions creates the circumstances for health conditions to cluster and, consequently, increases health inequalities among individuals and populations.^3^,^5^ This phenomenon is called a syndemic, which postulates that contextual conditions encourage the clustering of health conditions. When two or more diseases or health conditions cluster, they may interact, leading to more adverse health outcomes in individuals and populations than would be expected from having just one condition.^6^,^10^ Nevertheless, current healthcare systems and interventions focusing on adolescent health are designed to treat single diseases or health conditions, putting adolescents with multimorbidity or syndemics at risk of inefficient, contradictory or duplicative services.^1 11^

Although syndemic research in adolescent populations is limited, some studies have described multimorbidity and the effects of social contextual factors on adolescents’ health. Mental–physical multimorbidity is often described as a prevalent issue in the adolescent population.^12^,^15^ For example, anxiety, mood disorders and attention-deficit/hyperactivity disorder (e.g., ADHD) tend to co-occur with asthma, obesity, allergies and epilepsy.^14^

Social contextual factors, operating on the individual, household and parental levels, may act as risk and/or protective factors for the occurrence of health conditions, explaining why some individuals are more vulnerable to adverse health outcomes than others.^3 16^ For example, research shows that early exposure to parental stress related to finances, work or income is associated with poor health outcomes or psychological distress later in life.^5 17 18^ Additionally, parental physical health predominantly affects the overall health of children aged 10–12, while parental mental health is a more important factor for children aged 13–15.^19^

A unique syndemic study in Western Europe by Slagboom *et al.* provides narrative evidence for the presence of syndemic vulnerability within families and across generations, suggesting that syndemic vulnerability might manifest at a young age.^5^ However, adolescent multimorbidity studies and syndemic research often focus on adults or specific (clinical) populations or lack generalisability due to a small age range and a limited set of included health conditions.^20^,^27^ Additionally, much of the syndemic research has been conducted in developing, low-income or middle-income countries.^28^,^31^

Despite the Netherlands being known as a generous welfare state with organised care, significant social and health inequalities continue to exist between neighbourhoods in urban areas.^32^ Using a syndemic approach to explore multimorbidity in relation to social contextual factors among adolescents can improve our understanding of the mechanisms that potentially contribute to social and health inequalities. This approach informs an integrated strategy that combines health, social care and preventative activities to address health inequalities at an early age and promote efficient healthcare. Therefore, this study:

1. Explores the existence of clustered health conditions among adolescents aged 10–19, registered at primary care practices in the Dutch urban cities of The Hague and Leiden.
2. Identifies characteristics at the individual, household and parental level associated with clusters of health conditions.
3. Compares healthcare use and costs between adolescents with clustered health conditions, a single condition and no conditions.

Our findings advance our understanding of adolescents’ vulnerability to syndemics, and whose multimorbidity may be likelier to persist into adulthood, causing lower well-being and worse health.

## Methods

This cross-sectional population-based study is part of the project ‘Countering syndemic vulnerability: A community resilience approach’. The project aims to develop a community resilience approach in vulnerable neighbourhoods in the Netherlands, employing syndemic theory. This substudy contributes to mapping the syndemic vulnerability in the participating cities within the project.

For this study, a unique dataset was used, integrating routinely registered primary care records of general practitioners (GPs) connected to the Extramural Leiden University Medical Centrum Academic Network (ELAN) primary care network, and societal data routinely collected by the System of Social Statistical Datasets (SSD) from Statistics Netherlands (SN). ELAN provides medical patient data collected in the primary care setting in the Leiden and The Hague region.^33^ It includes information on GP-registered disease episodes and the period of concern regarding a health issue, coded according to the WHO International Classification of Primary Care (ICPC). The SSD consists of routinely collected individual-level societal data, including sociodemographic, socioeconomic and household characteristics.^34^ Data from both sources were coded, anonymised, stored securely within the SN environment and linked through anonymised individual ID numbers.

### Study population

The study population comprised all adolescents registered with a participating GP and living in two Dutch cities, The Hague and Leiden. The Hague is one of the largest cities in The Netherlands with more residents with a migration background, a lower average socioeconomic status (SES) score and a smaller prevalence of residents with a good self-perceived health compared with Leiden.^35 36^ Both cities exhibit disparities in health outcomes and SES scores across neighbourhoods. However, the city of The Hague shows larger differences in SES scores between neighbourhoods.^37^ Following the WHO, adolescents were defined as being 10–19 years old as of 1 January 2018. In the Netherlands, all residents are registered with a GP, usually in their neighbourhood, making the GP-registered population an adequate representation of the adolescent population living in both cities. Our study population consisted of all adolescents aged 10–19 years who were registered with a participating GP in one of the cities for at least 12 months during 2018–2019 and were alive throughout both years. We determined an individual’s residence in The Hague or Leiden based on their longest registered address in 2018.

### Patient and public involvement

No patients or members of the public were involved in formulating the research question, selecting the outcome measures, or in the development of the design or implementation of the study.

### Health conditions

The selection of health conditions in our study was based on prevalent health conditions in adolescents, prior research on comorbidity and multimorbidity in adolescents, and the available data.^1 11 38^ In this study, we use the term ‘health conditions’ to define chronic health conditions or health conditions that have a long-lasting effect. We subdivided health conditions into physical (e.g., asthma, cancer and epilepsy), mental (e.g., externalising behavioural disorders, such as ADHD, and internalising disorders, such as anxiety and feeling depressed) and psychosomatic (e.g., generalised fatigue/pain and headache disorders) health conditions (online supplemental appendix 1).

In the Netherlands, GPs register symptoms and diagnoses of health conditions as episodes using the ICPC. We identified the presence of health conditions through the ICPC codes related to both symptoms and diagnosis. The 2-year prevalence of these conditions was determined based on whether an individual had registered an active, new or mutated episode of the included ICPC codes in either 2018 or 2019. Adolescents were categorised as having none of the included health conditions (no morbidity), having one of the conditions (single morbidity), or having two or more of the conditions (multimorbidity) across the 2-year period in 2018–2019.

### Social contextual factors

We included a wide range of social contextual factors at the individual, household and parental levels (online supplemental appendix 2). These factors were selected for their known long-term impact on health and multimorbidity, based on prior research and available data.^5 11 17 18 20^

#### Individual-level factors

The SSD includes a diverse set of individual-level data, such as age, sex, country of origin (based on the individual’s and their parent’s birthplace), and whether the individual was a juvenile crime suspect, a victim of a criminal act, or had dropped out of school early between 2015 and 2019. From ELAN, we included information about adolescents having social problems, as registered by a GP in 2018 and 2019.

#### Household-level factors

At the household level, we extracted data about household composition (e.g., a single parent household and household composition) in 2018 and socioeconomic characteristics, such as household income in 2018 and SES of the neighbourhood where the individual was living in 2019. Information regarding adolescents in contact with Dutch youth protection services due to concerns about an unsafe family environment from 2015 to 2019 was also extracted.

#### Parental-level factors

To extract information at the parental level, a unique identification code enabled the linkage of adolescents to their parents. We incorporated information about the highest achieved education level attained by the parents, whether parents had debts, had been imprisoned, were victims of a criminal act or got divorced between 2015 and 2019. ELAN provided information on parents’ physical (e.g., cancer, hypertensive heart disease, stroke), mental (eg, burnout, mood disorders, hyperactivity disorders), and somatic (e.g., headache, generalised fatigue/pain) health conditions as well as their social problems between 2016 and 2019.

#### Healthcare use and costs

Adolescents’ GP healthcare use, healthcare costs and contacts with youth care were included as indications of the severity of the clustered health conditions. Variables related to GP healthcare use were derived from the total number of GP visits made within 2018 and 2019. The GP is the first healthcare provider a patient visits within the primary care system, without the need for a referral. The GP evaluates the severity of the patient’s complaint, provides treatment, prescribes medication and, when necessary, refers the patient to a secondary care specialist, which requires a formal referral. In the Netherlands, GPs function as gatekeepers, meaning that patients must see a GP before accessing specialised care. They operate in accordance with established guidelines for managing health conditions and adhere to professional standards. While these guidelines provide some consistency in care, the frequency of follow-up visits may still vary depending on the individual patient’s needs.

Healthcare costs included all expenses incurred by the care-using adolescent associated with the Health Insurance Act (e.g., GP costs and hospital care costs), excluding youth care costs, and were calculated as the average healthcare costs in euros from 2018 to 2019. The lowest possible maximum personal contribution towards healthcare costs in the Netherlands is €385 per year, which does not apply to children under 18 years of age.

Adolescents’ contact with youth care in 2018 and 2019 was set as a dichotomous variable. ‘Yes’ indicated involvement in one or more forms of assistance or care under the Youth Act, excluding youth protection and youth rehabilitation, such as support for psychological or parenting problems.

### Statistical analysis

The data were prepared using IBM SPSS Statistics V.25. Statistical analyses were conducted using RStudio (V.4.2.3). Descriptive statistics were used to describe the characteristics of adolescents with no morbidity, single morbidity and multimorbidity.

#### Latent class analysis

Latent class analysis (LCA) was conducted using the ‘poLCA’ package to explore different multimorbidity classes in the total adolescent population aged 10–19 with multimorbidity. This technique was chosen for its ability to group individuals without prior assumptions about the number and nature of the latent classes. The findings of Nichols *et al.* suggest that low-prevalence diseases may introduce noise into the modelling process.^39^ Consequently, in the present study, only conditions with a prevalence of at least 5% in the multimorbidity population were included in the analysis.

The LCA was conducted without a priori assumptions about the number of latent classes. We assessed model fit based on fit statistics, the interpretability of classes and the models’ entropy for clearer class separation (online supplemental table 1).^40^ The prevalence of health conditions within each class was derived from the posterior probability of the condition being present and individuals were assigned to the class with the highest predicted class membership (based on posterior probabilities).

#### Multinomial logistic regressions

Descriptive statistics were used to describe the presence of individual, household and parental characteristics across the latent classes. Multivariable multinomial logistic regression analyses were performed to explore the association between class memberships and individual, household and parental characteristics and to examine the association between memberships and GP healthcare use and costs. Finally, binary logistic regression analyses were performed to examine the association between class membership and contact with youth care.

The significance level for all regression analyses was set at p<0.05.

## Results

The total study population consisted of 10 841 adolescents (table 1). About half of the adolescents were aged 10–14 years (54.9%) and were male (50.5%). The majority lived in The Hague (81.1%) and 45.1% were of Dutch origin (table 1). In total, 2781 (25.7%) adolescents were registered as having at least two health conditions during the study period, indicating multimorbidity.

**Table 1:** Sociodemographic and social contextual characteristics of the study population sorted by morbidity groups*

	No morbidity	Single morbidity	Multimorbidity	Total study population	P value†
	n=4780 (44.1%)	n=3280 (30.3%)	n=2781 (25.7%)	n=10 841 (100%)	
	n (%)	n (%)	n (%)	n (%)	
Age‡					
10–14 years	2797 (58.5)	1795 (54.7)	1362 (49.0)	5954 (54.9)	<0.001
15–19 years	1983 (41.5)	1485 (45.3)	1419 (51.0)	4887 (45.1)	
Sex					
Male	2600 (54.4)	1601 (48.8)	1274 (45.8)	5475 (50.5)	<0.001
Female	2180 (45.6)	1679 (51.2)	1507 (54.2)	5366 (49.5)	
Country of origin					
The Netherlands	2113 (44.2)	1503 (45.8)	1268 (45.6)	4884 (45.1)	<0.001
Europe (excl. The Netherlands)	427 (8.9)	240 (7.3)	192 (6.9)	859 (7.9)	
Turkey/Morocco	971 (20.3)	765 (23.3)	653 (23.5)	2389 (22.0)	
Suriname/Indonesia/Dutch Caribbean	514 (10.8)	352 (10.7)	314 (11.3)	1180 (10.9)	
Other (Africa/Asia/America/Oceania)	755 (15.8)	420 (12.8)	354 (12.7)	1529 (14.1)	
Juvenile crime suspect§					
No	4633 (96.9)	3147 (95.9)	2677 (96.3)	10 607 (97.8)	<0.01
Yes	147 (3.1)	133 (4.1)	132 (4.7)	412 (3.8)	
School dropout§					
No	4588 (96.0)	3126 (95.3)	2569 (92.4)	10 283 (94.9)	<0.001
Yes	192 (4.0)	154 (4.7)	212 (7.6)	558 (5.1)	
Victim of a criminal act§					
No	4491 (94.0)	3007 (91.7)	2459 (88.4)	9957 (91.8)	<0.001
Yes	289 (6.0)	273 (8.3)	322 (11.6)	884 (8.3)	
Social problems¶					
No	4597 (96.2)	3101 (94.5)	2549 (91.7)	10 247 (94.5)	<0.001
Yes	183 (3.8)	179 (5.5)	232 (8.3)	594 (5.5)	
Single parent household‡					
No	3607 (75.5)	2469 (75.3)	1949 (70.1)	8025 (74.0)	<0.001
Yes	1173 (24.5)	811 (24.7)	832 (29.9)	2816 (26.0)	
Household composition‡					
4 or less	2963 (62.0)	2144 (65.4)	1948 (70.0)	7055 (65.1)	<0.001
More than 4	1817 (38.0)	1136 (34.6)	833 (30.0)	3786 (34.9)	
Known at youth protection services¶					
No	4714 (98.6)	3216 (98.0)	2677 (96.3)	10 607 (97.8)	
Yes	66 (1.4)	64 (2.0)	104 (3.7)	234 (2.2)	<0.001
Household income‡					
Low	702 (14.7)	445 (13.6)	436 (15.7)	1583 (14.6)	<0.001
Moderate	3177 (66.5)	2394 (73.0)	2005 (72.1)	7576 (69.9)	
High	786 (16.4)	390 (11.9)	270 (9.7)	1446 (13.3)	
Other	115 (2.4)	51 (1.6)	70 (2.5)	236 (2.2)	
Socioeconomic status neighbourhood**					
Low	2539 (53.1)	1766 (53.8)	1473 (53.0)	5778 (53.3)	<0.001
Average	446 (9.3)	248 (7.6)	192 (6.9)	886 (8.2)	
High	1794 (37.5)	1266 (38.6)	1116 (40.1)	4176 (38.5)	
Parent(s) having debts§					
No	4323 (90.4)	2958 (90.2)	2438 (87.7)	9719 (89.7)	−0.001
Yes	457 (9.6)	322 (9.8)	343 (12.3)	1122 (10.3)	
Parent(s) being detained or suspect of a crime§					
No	4421 (92.5)	2993 (91.3)	2501 (89.9)	9915 (91.5)	0.002
Yes	359 (7.5)	287 (8.8)	280 (10.1)	926 (8.5)	
Parent(s) being victim of a criminal act§					
No	3867 (80.9)	2599 (79.2)	2186 (78.6)	8652 (79.8)	0.082
Yes	913 (19.1)	681 (20.8)	595 (21.4)	2189 (20.2)	
Parent(s) being divorced§					
No	4274 (89.4)	2906 (88.6)	2482 (89.2)	9662 (89.1)	0.706
Yes	506 (10.6)	374 (11.4)	299 (10.8)	1179 (10.9)	
Highest achieved educational level parent(s)††					
Low	1148 (24.0)	858 (26.2)	818 (29.4)	2824 (26.0)	<0.001
Middle	842 (17.6)	676 (20.6)	629 (22.6)	2147 (19.8)	
High	1985 (41.5)	1263 (38.5)	949 (34.1)	4197 (38.7)	
Missing	805 (16.8)	483 (14.7)	385 (13.8)	1673 (15.4)	
Parent(s) with a chronic physical health condition‡‡					
None	1847 (12.1)	847 (25.8)	473 (17.0)	3167 (29.2)	<0.001
One parent	2181 (29.8)	1611 (49.1)	1441 (51.8)	5233 (48.3)	
Both parents	752 (28.1)	822 (25.1)	867 (31.2)	2441 (22.5)	
Parent(s) with a mental disorder‡‡					
None	2977 (62.3)	1544 (47.1)	1021 (36.7)	5542 (51.1)	<0.001
One parent	1525 (31.9)	1400 (42.7)	1316 (47.3)	4241 (39.1)	
Both parents	278 (5.8)	336 (10.2)	444 (16.0)	1058 (9.8)	
Parent(s) with a somatic health condition‡‡					
None	2048 (42.8)	998 (30.4)	686 (24.7)	3732 (34.4)	<0.001
One parent	2090 (43.7)	1649 (50.3)	1465 (52.7)	5204 (48.0)	
Both parents	642 (13.4)	633 (19.3)	630 (22.7)	1905 (17.6)	
Parent(s) with social problems‡‡					
No	3937 (82.4)	2490 (75.9)	1924 (69.2)	8351 (77.0)	<0.001
Yes	843 (17.6)	790 (24.1)	857 (30.8)	2490 (23.0)	
GP healthcare use¶					
No visits	2953 (61.8)	1004 (30.6)	353 (12.7)	4310 (39.8)	<0.001
1–3 visits	1301 (27.2)	1229 (37.5)	754 (27.1)	3284 (30.3)	
4–6 visits	388 (8.1)	654 (19.9)	699 (25.1)	1741 (16.1)	
More than 6 visits	138 (2.9)	393 (12.0)	975 (35.1)	1506 (13.9)	
Healthcare costs¶					
Through 385	2438 (51.0)	1236 (37.7)	642 (23.1)	4316 (39.8)	<0.001
385–2000	2097 (43.9)	1769 (53.9)	1698 (61.1)	5564 (51.3)	
2000 or more	213 (4.5)	269 (8.2)	438 (15.7)	920 (8.5)	
Missing	32 (0.7)	6 (0.2)	3 (0.1)	41 (0.4)	
In contact with youth care§					
No	3707 (77.6)	2223 (67.8)	1424 (51.2)	7354 (67.8)	<0.001
Yes	1073 (22.4)	1057 (32.2)	1357 (48.8)	3487 (32.2)	

* A detailed overview of the definitions and categories of the variables is provided in online supplemental appendix 2.

† ANOVA test was performed on continuous variables, and a χ² test on categorical variables.

‡ 2018.

§ 2015–2019.

¶ 2018–2019.

** 2019.

†† 2015–2018.

‡‡ 2016–2019.

ANOVA, analysis of variance; GP, general practitioner.

Table 2 shows the 2-year prevalence of health conditions among the single, multimorbidity and total population groups. We included the 11 most frequent health conditions in the multimorbidity group in the LCA analysis, namely (chronic) neck and back conditions (21.1%), headache disorders (20.9%), asthma (21.9%), gastrointestinal conditions (30.2%), generalised fatigue/pain (23.4%), skin conditions (45.3%), overweight/obesity (12.4%), (head) trauma and fractures (16.3%), externalising behavioural disorders (including ADHD) (20.6%), internalising disorders (21.8%) and burnout/stress (10.7%).

**Table 2:** A 2-year prevalence of health conditions in 2018 and 2019 sorted by presence of single and multimorbidity*

	Single morbidity	Multimorbidity	Total study population
	n=3280	n=2781	n=10 841
	n (%)†	n (%)†	n (%)†
Endocrine disorders	27 (0.8)	71 (2.6)	98 (0.9)
Cancer	11 (0.3)	29 (1.0)	40 (0.4)
Cardiac disease	13 (0.4)	22 (0.8)	35 (0.3)
(Chronic) neck and back conditions	253 (7.7)	**587** (**21.1**)	840 (7.7)
Headache disorders	182 (5.5)	**581** (**20.9**)	763 (7.0)
Asthma	259 (7.9)	**608** (**21.9**)	867 (8.0)
Gastrointestinal conditions	367 (11.2)	**841** (**30.2**)	1208 (11.1)
Generalised fatigue/pain	291 (8.9)	**651** (**23.4**)	942 (8.7)
Skin conditions	797 (24.3)	**1259** (**45.3**)	2056 (19.0)
Overweight/obesity	106 (3.2)	**344** (**12.4**)	450 (4.2)
(Head) trauma and fractures	209 (6.4)	**454** (**16.3**)	663 (6.1)
Epilepsy	23 (0.7)	53 (1.9)	76 (0.7)
Sexual behaviour	30 (0.9)	50 (1.8)	80 (0.7)
Externalising behavioural disorders	300 (9.1)	**574** (**20.6**)	874 (8.1)
Internalising disorders	241 (7.3)	**607** (**21.8**)	848 (7.8)
Burnout/stress	116 (3.5)	**297** (**10.7**)	413 (3.8)
Suicide attempts		20 (0.7)	
Substance abuse	31 (0.9)	78 (2.8)	109 (1.0)
Other mental disorders	19 (0.6)	95 (3.4)	114 (1.1)

The conditions >5% in the multimorbidity group are included as indicator variables in the LCA and shown in bold*.*

* Overview of the ICPC of the health conditions can be found in online supplemental appendix 1.

† Proportions were calculated by using the total number of adolescents in the corresponding subgroup as the denominator.

ICPC, International Classification of Primary Care; LCA, latent class analysis.

### LCA class interpretation

We selected the four-class model, considering the fit indices and interpretability, with an entropy of 0.91 (online supplemental table 1). Although the Akaike information criterion (AIC) continued to decrease slightly as the number of classes was increased, suggesting a more significant number of classes as an optimal fit, the AIC tends to overfit complex models.^40^

Table 3 presents class proportions and the probability prevalence of health conditions within each class (online supplemental figure 1). Classes were labelled based on the health conditions with the highest probability prevalence within each class and those with striking prevalence differences compared with the other classes and the total study population. The four classes were:

**Table 3:** Probability prevalence rates of health conditions by the four-multimorbidity classes* identified by LCA

	Class 1	Class 2	Class 3	Class 4
	Asthma-skin conditions	Skin conditions-pain	Externalising behavioural disorders and skin conditions	Gastrointestinal conditions, internalising disorders and pain
Class membership probabilities (%)	14.7	29.2	18.2	37.9
Probability prevalence based on the LCA† (%)	15.9	28.6	20.3	35.2
(Chronic) neck and back conditions	11.9	**20.2**	13.3	**29.1**
Headache disorders	14.5	**21.7**	13.1	**26.6**
Asthma	**100**	0	**21.2**	8.8
Gastrointestinal conditions	**20.2**	**27.9**	18.7	**41.5**
Generalised fatigue/pain	10.6	**21.2**	17.1	**33.2**
Skin conditions	**60.3**	**100**	**39.5**	0
Overweight/obesity	10.5	12.1	8.9	15.0
(Head) trauma and fractures	8.9	13.4	16.6	**21.4**
Externalising behavioural disorders	0	2.3	**100**	4.6
Internalising disorders	11.7	19.0	12.9	**32.2**
Burnout/stress	6.3	10.3	9.0	13.5

Bold numbers indicate conditions with a class prevalence of ≥20% (by column).

* Individuals were assigned to a latent class based on the highest posterior probability for each latent class.

† Prevalence is based on the posterior probability that the health condition is present in the latent class.

LCA, latent class analysis.

#### Class 1: ‘asthma–skin conditions’ (n=442, 15.9% in multimorbidity group, 4.1% in total study population)

The first multimorbidity class had the lowest prevalence of adolescents, with 4.1% in the total study population. This class was characterised by the highest likelihood of children having asthma, combined with a high likelihood of skin conditions and gastrointestinal conditions.

#### Class 2: ‘skin conditions–pain’ (n=794, 28.6% in multimorbidity group, 7.3% in total study population)

The second multimorbidity class had a prevalence of 7.3% in the total study population and was characterised by the highest likelihood of skin conditions. Additional conditions with a high probability prevalence were gastrointestinal conditions, headache disorders, generalised fatigue/pain, and (chronic) neck and back conditions.

#### Class 3: ‘externalising behavioural disorders and skin conditions’ (n=565, 20.3% in multimorbidity group, 5.2% in total study population)

Of all children included in this study, a prevalence of 5.2% was categorised into the third multimorbidity class. Externalising behavioural disorders were the primary health condition in this class having the highest probability prevalence, followed by a high likelihood of skin conditions and asthma.

#### Class 4: ‘gastrointestinal conditions, internalising disorders and pain’ (n=980, 35.2% in multimorbidity group, 9.0% in study population)

The fourth class had the highest class prevalence of 9.0% in the total study population. Unlike other classes, class 4 does not distinguish one specific health condition. However, compared with other classes, gastrointestinal conditions, generalised fatigue/pain, internalising disorders, (chronic) neck and back conditions, headache disorders and (head) trauma and fractures had the highest likelihood among adolescents in class 4.

### Social contextual factors

Table 4 shows the associations (OR(95% CI)) between social contextual factors and the multimorbidity classes based on the multinomial logistic regression with the no morbidity group as the reference category.

**Table 4:** Associations between social contextual factors and multimorbidity classes

	Single morbidity	Class 1	Class 2	Class 3	Class 4
	n=3280 (30.3%)	n=442 (4.1%)*	n=794 (7.3%)*	n=565 (5.2%)*	n=980 (9.0%)*
	OR (95% CI)	OR (95%CI)	OR (95% CI)	OR (95% CI)	OR (95% CI)
Intercept	**0.27 (0.21 to 0.34**)	**0.02 (0.01 to 0.03**)	**0.03 (0.02 to 0.05**)	**0.02 (0.01 to 0.04**)	**0.03 (0.02 to 0.04**)
Age					
10–14 years†	1.00	1.00	1.00	1.00	1.00
15–19 years	1.09 (0.98 to 1.21)	1.16 (0.93 to 1.46)	1.04 (0.88 to 1.25)	**0.80 (0.64 to 0.98**)	1.81 (1.54 to 2.14)
Sex					
Male†	1.00	1.00	1.00	1.00	1.00
Female	**1.29 (1.16 to 1.42**)	1.11 (0.90 to 1.38)	**1.84 (1.55 to 2.19**)	**0.79 (0.65 to 0.97**)	2.08 (1.78 to 2.44)
Country of origin					
The Netherlands†	1.00	1.00	1.00	1.00	1.00
Europe (excl. The Netherlands)	0.92 (0.75 to 1.13)	0.84 (0.52 to 1.37)	1.10 (0.77 to 1.57)	0.87 (0.58 to 1.31)	1.16 (0.84 to 1.58)
Turkey/Morocco	1.01 (0.86 to 1.18)	1.17 (0.85 to 1.62)	1.13 (0.88 to 1.47)	**0.56 (0.40 to 0.77**)	1.10 (0.87 to 1.40)
Suriname/Indonesia/Dutch-Caribbean	0.95 (0.80 to 1.12)	0.90 (0.62 to 1.32)	1.08 (0.81 to 1.44)	0.77 (0.56 to 1.06)	0.89 (0.68 to 1.17)
Other (Africa/Asia/America/Oceania)	0.87 (0.74 to 1.03)	1.07 (0.76 to 1.50)	0.88 (0.66 to 1.17)	0.83 (0.61 to 1.14)	0.90 (0.70 to 1.17)
Juvenile crime suspect					
No†	1.00	1.00	1.00	1.00	1.00
Yes	1.29 (0.98 to 1.70)	1.34 (0.78 to 2.31)	0.77 (0.45 to 1.32)	**1.55 (1.01 to 2.38**)	1.26 (0.86 to 1.84)
School dropout					
No†	1.00	1.00	1.00	1.00	1.00
Yes	1.01 (0.78 to 1.30)	1.13 (0.68 to 1.86)	1.03 (0.67 to 1.57)	**1.79 (1.21 to 2.64**)	**1.56 (1.15 to 2.13**)
Victim of a criminal act					
No†	1.00	1.00	1.00	1.00	1.00
Yes	**1.27 (1.04 to 1.54**)	0.86 (0.55 to 1.35)	**1.80 (1.34 to 2.41**)	**2.08 (1.53 to 2.83**)	**1.72 (1.33 to 2.23**)
Social problems					
No†	1.00	1.00	1.00	1.00	1.00
Yes	1.13 (0.89 to 1.44)	1.44 (0.92 to 2.25)	**1.79 (1.29 to 2.48**)	**1.15 (0.78 to 1.70**)	**1.55 (1.14 to 2.12**)
Single parent household					
No†	1.00	1.00	1.00	1.00	1.00
Yes	0.96 (0.84 to 1.10)	**1.37 (1.04 to 1.81**)	1.09 (0.87 to 1.35)	**1.52 (1.19 to 1.93**)	1.13 (0.92 to 1.38)
Household composition					
4 or less†	1.00	1.00	1.00	1.00	1.00
More than 4	**0.86 (0.77 to 0.97**)	0.85 (0.67 to 1.10)	**0.73 (0.60 to 0.90**)	**0.65 (0.51 to 0.82**)	**0.70 (0.58 to 0.84**)
Known at Youth Protection Services					
No†	1.00	1.00	1.00	1.00	1.00
Yes	1.35 (0.92 to 1.99)	0.73 (0.28 to 1.93)	1.37 (0.74 to 2.53)	**1.90 (1.12 to 3.22**)	**2.43 (1.54 to 3.82**)
Household income					
Low†	1.00	1.00	1.00	1.00	1.00
Moderate	**1.22 (1.05 to 1.41**)	1.20 (0.88 to 1.64)	1.06 (0.83 to 1.35)	1.06 (0.79 to 1.41)	1.21 (0.96 to 1.52)
High	0.88 (0.71 to 1.10)	0.92 (0.57 to 1.49)	**0.71 (0.48 to 1.03**)	0.74 (0.48 to 1.14)	1.04 (0.74 to 1.47)
Other	0.92 (0.59 to 1.45)	1.87 (0.82 to 4.25)	0.58 (0.24 to 1.43)	**2.00 (1.07 to 3.76**)	1.43 (0.81 to 2.52)
SES neighbourhood					
Low†	1.00	1.00	1.00	1.00	1.00
Average	0.88 (0.73 to 1.07)	1.19 (0.80 to 1.77)	0.84 (0.59 to 1.20)	0.83 (0.55 to 1.24)	0.81 (0.58 to 1.12)
High	1.13 (1.00 to 1.28)	1.23 (0.94 to 1.60)	**1.33 (1.08 to 1.63**)	**1.79 (1.42 to 2.26**)	**1.35 (1.11 to 1.63**)
Parent(s) having debts					
No†	1.00	1.00	1.00	1.00	1.00
Yes	0.95 (0.80 to 1.13)	0.87 (0.60 to 1.25)	0.92 (0.69 to 1.22)	1.15 (0.85 to 1.54)	1.10 (0.85 to 1.41)
Parent(s) being detained or suspect of a crime					
No†	1.00	1.00	1.00	1.00	1.00
Yes	1.00 (0.83 to 1.21)	0.82 (0.54 to 1.24)	0.91 (0.67 to 1.24)	1.26 (0.92 to 1.72)	0.94 (0.72 to 1.25)
Parent(s) being victim of a criminal act					
No†	1.00	1.00	1.00	1.00	1.00
Yes	1.00 (0.88 to 1.13)	0.80 (0.61 to 1.06)	0.90 (0.73 to 1.11)	1.02 (0.81 to 1.30)	0.99 (0.81 to 1.20)
Parent(s) being divorced					
No†	1.00	1.00	1.00	1.00	1.00
Yes	0.98 (0.83 to 1.16)	**0.61 (0.42 to 0.90**)	**0.73 (0.55 to 0.98**)	**0.57 (0.41 to 0.78**)	0.71 (0.54 to 0.92)
Highest achieved educational level parent(s)					
Low†	1.00	1.00	1.00	1.00	1.00
Middle	1.06 (0.92 to 1.23)	1.15 (0.86 to 1.54)	1.04 (0.83 to 1.30)	1.27 (0.98 to 1.66)	0.94 (0.76 to 1.17)
High	0.98 (0.85 to 1.13)	0.91 (0.68 to 1.23)	0.81 (0.64 to 1.02)	1.00 (0.76 to 1.31)	**0.78 (0.63 to 0.96**)
Parent(s) with a chronic physical health condition					
None†	1.00	1.00	1.00	1.00	1.00
One parent	**1.41 (1.25 to 1.59**)	**2.34 (1.73 to 3.16**)	**1.94 (1.54 to 2.44**)	**2.03 (1.55 to 2.64**)	**1.99 (1.62 to 2.45**)
Both parents	**1.82 (1.56 to 2.12**)	**3.39 (2.40 to 4.79**)	**3.47 (2.67 to 4.52**)	**2.97 (2.17 to 4.05**)	**2.72 (2.13 to 3.48**)
Parent(s) with a mental disorder					
None†	1.00	1.00	1.00	1.00	1.00
One parent	**1.49 (1.34 to 1.66**)	**1.52 (1.20 to 1.93**)	**1.64 (1.36 to 1.98**)	**2.21 (1.76 to 2.77**)	**1.83 (1.54 to 2.19**)
Both parents	**1.65 (1.36 to 2.01**)	**2.09 (1.46 to 2.99**)	**2.00 (1.49 to 2.67**)	**3.91 (2.84 to 5.38**)	**2.65 (2.03 to 3.46**)
Parent(s) with a somatic health condition					
None†	1.00	1.00	1.00	1.00	1.00
One parent	**1.32 (1.18 to 1.48**)	**1.36 (1.04 to 1.77**)	**1.47 (1.19 to 1.82**)	**1.55 (1.22 to 1.96**)	**1.50 (1.24 to 1.82**)
Both parents	**1.46 (1.25 to 1.72**)	**1.79 (1.29 to 2.50**)	**1.79 (1.37 to 2.34**)	**1.58 (1.15 to 2.17**)	**1.89 (1.47 to 2.42**)
Parent(s) with social problems					
No†	1.00	1.00	1.00	1.00	1.00
Yes	**1.17 (1.03 to 1.32**)	**1.44 (1.12 to 1.85**)	**1.51 (1.24 to 1.83**)	**1.81 (1.46 to 2.25**)	**1.27 (1.06 to 1.53**)

No morbidity: reference category

Bold numbers: p<0.005 for OR (95% CI).

A detailed overview of the definitions and categories of the variables is provided in online supplemental appendix 2.

n (%) characteristics of social contextual factors and multimorbidity classes are provided in online supplemental table 2.

* Class membership of individuals is based on the highest predicted probability of belonging to a class. Only the adolescents with determined multimorbidity were included in the LCA.

† Reference group.

LCA, latent class analysis; SES, socioeconomic status.

#### Individual-level factors

On the individual level, female adolescents had higher odds of belonging to class 2 (1.84 (95% CI 1.55 to 2.19)) and lower odds of belonging to class 3 (0.79 (95% CI 0.65 to 0.97)), compared with the no morbidity group. Looking at age, 15–19 years had lower odds of belonging to class 3 (0.80 (95% CI 0.64 to 0.98)). In addition, adolescents being born in Turkey or Morocco or with one or both parents or being born in these countries were less likely to belong to class 3 (0.56 (95% CI 0.40 to 0.77)).

Juvenile crime suspects were likelier to belong to class 3 (1.55 (95% CI 1.01 to 2.38)), and early school dropouts had higher odds of belonging to classes 3 (1.79 (95% CI 1.21 to 2.64)) or 4 (1.56 (95% CI 1.15 to 2.13)). Both victims of a known criminal act and adolescents with GP-registered social problems had higher odds of belonging to classes 2 (1.80 (95% CI 1.34 to 2.41)), 3 (2.08 (95% CI 1.53 to 2.83)) and 4 (1.72 (1.33 to 2.23)).

#### Household-level factors

Adolescents living in a single parent household had higher odds of belonging to classes 1 (1.37 (95% CI 1.04 to 1.81)) and 3 (1.52 (95% CI 1.19 to 1.93)), adolescents living in a household with more than four persons had lower odds of belonging to classes 2 (0.73 (95% CI 0.60 to 0.90)), 3 (0.65 (95% CI 0.51 to 0.82)) and 4 (0.70 (95% CI 0.58 to 0.84)). Adolescents who had contact with Dutch Youth Protective Services had higher odds of belonging to classes 3 (1.90 (95% CI 1.12 to 3.22)) and 4 (2.43 (95% CI 1.54 to 3.82)).

Adolescents with a high household income were less likely to belong to class 2 (0.71 (95% CI 0.48 to 1.03)), while adolescents with a household income defined as other, referring to individuals with an unknown household income or belonging to a student or institutionalised household, were likelier to belong to class 3 (2.00 (95% CI 1.07 to 3.76)). Adolescents living in a high-SES neighbourhood had higher odds of belonging to classes 2 (1.33 (1.08 to 1.63)), 3 (1.79 (1.42 to 2.26)) and 4 (1.35 (1.11 to 1.63)).

#### Parental-level factors

Compared with the no morbidity group, adolescents of parents with a higher education level had lower odds of belonging to class 4 (0.78 (95% CI 0.63 to 0.96)). All classes were less likely to have parents divorced in the past 5 years.

When adolescents had one or both parents with a GP-registered physical, mental, somatic health conditions, or with GP-registered social problems, odds on belonging to the group with one morbidity and to all four multimorbidity classes were higher compared with the no morbidity group.

### Healthcare use and costs

Table 5 shows both the prevalence of healthcare use and costs of all class memberships and the associations between the multimorbidity classes and healthcare use and costs.

**Table 5:** Associations between multimorbidity classes and healthcare use

GP healthcare use**	No visits*		1–3 visits		4–6 visits		More than 6 visits	
Class memberships	n (%)	OR (95% CI)	n (%)	OR (95% CI)	n (%)	OR (95% CI)	n (%)	OR (95% CI)
Intercept				0.44(0.41 to 0.47)		0.13(0.12 to 0.15)		0.05(0.04 to 0.06)
No morbidity	2953 (61.8)	1.00	1301 (27.2)	1.00	388 (8.1)	1.00	138 (2.9)	1.00
Single morbidity	1004 (30.6)	1.00	1229 (37.5)	2.78(2.50 to 3.09)	654 (19.9)	4.96(4.29 to 5.73)	393 (12.0)	8.38(6.81 to 10.30)
Class 1†	47 (10.6)	1.00	135 (30.5	6.52(4.65 to 9.14)	116 (25.3)	18.78(13.17 to 26.79)	144 (32.6)	65.56(45.25 to 95.00)
Class 2†	77 (9.7)	1.00	220 (27.7)	6.49(4.96 to 8.48)	198 (24.9)	19.57(14.74 to 25.99)	299 (37.7)	83.10(61.37 to 112.52)
Class 3†	58 (10.3)	1.00	176 (31.2)	6.89(5.08 to 9.33)	156 (27.6)	20.47(14.87 to 28.18)	175 (31.0)	64.57(45.84 to 90.95)
Class 4†	171 (17.4)	1.00	223 (22.8)	2.96(2.40 to 3.65)	229 (23.4)	10.19(8.14 to 12.76)	357 (36.4)	44.67(34.80 to 57.35)
Healthcare costs**	**Through 385** *		**385–2000**		**2000 or more**			**Missing**
**Class memberships**	**n (%)**	**OR (95% CI)**	**n (%)**	**OR (95% CI)**	**n (%)**	**OR (95% CI)**		**n (%)**
Intercept				0.86 (0.81 to 0.91)		0.09 (0.08 to 0.10)		
No morbidity	2438 (51.0)	1.00	2097 (43.9)	1	213 (4.5)	1		32 (0.7)
Single morbidity	1236 (37.7)	1.00	1769 (53.9)	1.66 (1.52 to 1.83)	269 (8.2)	2.49 (2.06 to 3.02)		6 (0.2)
Class 1†	117 (26.5)	1.00	263 (59.5)	2.61 (2.09 to 3.27)	61 (13.8)	5.97 (4.25 to 8.38)		32 (0.7)
Class 2†	186 (23.4)	1.00	486 (61.2)	3.04 (2.54 to 3.63)	121 (15.2)	7.45 (5.69 to 9.74)		1 (0.1)
Class 3†	132 (23.4)	1.00	356 (63.0)	3.14 (2.55 to 3.86)	77 (13.6)	6.68 (4.88 to 9.14)		0 (0)
Class 4†	207 (21.1)	1.00	593 (60.5)	3.33 (2.81 to 3.94)	179 (18.3)	9.90 (7.75 to 12.64)		1 (0.1)
In contact with youth care**	**No** *		**Yes**					
**Class memberships**	**n (%)**	**OR (95% CI)**	**n (%)**	**OR (95% CI)**				
Intercept				0.29 (0.27 to 0.31)				
No morbidity	3707 (77.6)	1.00	1073 (22.4)	1.00				
Single morbidity	2223 (67.8)	1.00	1057 (32.2)	1.64 (1.49 to 1.82)				
Class 1†	306 (67.8)	1.00	136 (30.8)	1.54 (1.24 to 1.90)				
Class 2†	485 (61.1)	1.00	309 (38.9)	2.20 (1.88 to 2.58)				
Class 3†	113 (20.0)	1.00	452 (80.0)	13.82 (11.12 to 17.17)				
Class 4†	520 (53.1)	1.00	460 (46.9)	3.06 (2.65 to 3.52)				

A detailed overview of the definitions and categories of the variables is provided in online supplemental appendix 2.

*p<0.05.

**p<0.001.

* Reference group.

† Class membership of individuals is based on the highest predicted probability of belonging to a class.

GP, general practitioner.

#### GP healthcare use

Compared with the no and single morbidity groups, all multimorbidity classes had higher odds of having more than 6 GP visits. Class 2 had the highest prevalence (37.7%) followed by class 4 (36.4%).

#### Healthcare costs

Compared with the no and single morbidity group, all the multimorbidity classes had higher odds of having healthcare costs of 2000 or more euros. Class 4 (gastrointestinal conditions, internalising disorders and pain) had the highest prevalence (18.3%), followed by class 2 (15.2%).

#### Youth care

Classes 2 (2.20 (95% CI 1.88 to 2.58)), 3 (13.82 (95% CI 11.12 to 17.17)) and 4 (3.06 (95% CI 2.65 to 3.52)), had higher odds of being in contact with youth care between 2015 and 2019, in comparison with the no and single morbidity group. Class 3 had the highest prevalence with more than half of the adolescents in this class having had contact with youth care.

## Discussion

The objective of this study was to explore whether, and if so, which clusters of health conditions are prevalent among adolescents aged 10–19 registered at primary care practices in the Dutch cities The Hague and Leiden. Additionally, we aimed to identify contributing social contextual factors and the association of multimorbidity classes with healthcare use and costs. One-quarter of the adolescents were registered as having at least two or more chronic health conditions in 2018 and 2019. To explore clusters of health conditions, these adolescents with multimorbidity were grouped based on probability of each individual fitting into a specific group using LCA. This method allowed for the identification of four distinct multimorbidity classes of health conditions, respectively class 1 (asthma–skin conditions), class 2 (skin conditions–pain), class 3 (externalising behavioural disorders and skin conditions) and, most prevalent, class 4 (gastrointestinal conditions, internalising disorders and pain). Skin conditions were a prevalent diagnosis in three of the classes. The fourth cluster of health conditions did not include skin disease and seemed to have a more psychosomatic nature.

Increased healthcare use and costs across all classes indicate a higher disease burden among adolescents with clustered health conditions, which is consistent with previous literature.^41^ Adolescents with gastrointestinal conditions, internalising disorders and pain (class 4) had the highest healthcare costs and use. Those with externalising behavioural disorders and skin conditions (class 3), however, most frequently used youth care. These two multimorbidity classes, 3 and 4, were characterised by several more prominent adverse social contextual factors.

The co-occurrence of physical and mental health conditions, along with adverse social conditions, emphasises the relevance of a syndemic approach. Syndemic theory posits that two or more clustered diseases or health conditions can interact within adverse social contextual conditions, leading to more adverse health outcomes.^6^,^10^ The disease clustering of both externalising and internalising disorders with other (somatic) health conditions is correlated with higher healthcare use and costs compared with adolescents having only one health condition. Moreover, these clustered conditions co-occur with pronounced adverse social contextual factors, such as an increased likelihood of school dropout.

Importantly, addressing adolescents’ syndemic vulnerability may require an intergenerational syndemic approach, as clustered health and social conditions were found to be passed down through generations. This demonstrates that syndemic vulnerability is potentially intergenerational.^5^ Our study showed that a large majority of the adolescents with multimorbidity also had one or both parents with physical, mental and/or somatic health conditions. These findings raise questions about the genetic, biological, social and/or behavioural transmittance of health conditions within families.

Evidence indicates intergenerational correlations or transmission of physical and mental health between parents and their offspring.^42 43^ Children of parents having a severe disease are at higher risk of developing mental health disorders and of reporting lower self-rated psychosomatic health and life satisfaction, scoring even lower when children themselves had a chronic disease.^44 45^ Due to the limited number of syndemic studies, our understanding of social contextual risk factors, intergenerational transmission and long-term effects is significantly less comprehensive compared with our knowledge of individual health conditions, highlighting the need for more attention to this clustering of health condition and social contextual circumstances in healthcare. Integrated preventative measures focusing on multi-level aspects are essential to reduce potential syndemic vulnerabilities. One recommended strategy is comprehensive prevention, a broad context-sensitive approach that considers overlapping and interacting adversities.^46^ A context-sensitive approach, combined with long-term family interventions targeting multiple generations—such as the three-generation approach—can enhance the health of parents, their child and following generations.^47^ Moreover, a Dutch study, using similar data, showed the early onset of health problems among children aged 0–12 years growing up in adverse or unfavourable social contexts, emphasising the importance of preventive measures from an early age.^48^

In line with our findings, other adolescent multimorbidity studies show similar multimorbidity classes.^1549^,^53^ Multiple studies have reported on the co-occurrence of asthma and skin conditions.^51 52^ Skin conditions, externalising behavioural disorders and pain complaints, such as stomach pain and headaches, are also common disease clusters in the literature,^49 50 54^ as are asthma and externalising disorders.^55 56^ These clusters of health conditions are often explained as resulting from mutually interacting biological or physical mechanisms.^49^,^5355^ Additionally, the biological mechanism of stress has been described as a potentially important factor in both skin diseases and externalising behavioural disorders.^57 58^

In line with the literature, the combination of skin diseases and pain was more prevalent among girls, and the combination of skin conditions and externalising behavioural disorders was more prevalent among boys. In our study, the latter group was also younger, less often included adolescents from Moroccan or Turkish origin and more often lived in a high SES neighbourhood. Cultural differences in parents’ recognition of externalising behavioural disorders may explain these findings, as a Dutch study reported lower problem recognition among parents of Moroccan or Turkish origin.^59^

Adolescents with externalising behavioural disorders were most clearly characterised by their increased use of youth care, emphasising the psychosocial aspect of this multimorbidity. They had an increased likelihood of having dropped out of school, of being a victim of a criminal act or being a juvenile crime suspect, of growing up in a single parent household or in a household that had contact with child protection services, and of having parents with GP-registered social problems. In short, social circumstances are central to this group of adolescents.

Parental stress arising from social circumstances in the family setting may affect the early development of mental health problems.^45 60^ Children who grow up under parental stress, in an unstable family environment, or experiencing parental divorce are at increased risk of developing problems in internalising or externalising behaviour during adolescence.^45^ Conversely, parental stress is shown to increase when children show externalising behaviour problems.^52^ For instance, in children with autism spectrum disorders, parental stress affects internalising and externalising behaviour problems, indicating a parent-driven effect. For externalising behaviours, effects are bidirectional, as the externalising behavioural problems of the child lead to increased parental stress, particularly among fathers.^61^ Additionally, these social and family problems, which contribute to externalising behavioural disorders, can increase the risks of school dropout and criminal behaviour.^62^,^64^ For example, adolescents living with a single parent may experience emotional distress when comparing themselves with peers in culturally preferred two-parent families.^62^

The psychosomatic nature of class 4 combined internalising disorders with gastrointestinal conditions and chronic pains, such as generalised fatigue/pain, neck and back conditions and headache disorders. Studies have shown that internalising disorders can be expressed as psychosomatic pain complaints, such as neck and back pain or headaches.^14^ Although it is not always clear whether internalising disorders and various pain complaints manifest with a similar aetiology or mutually interact,^65^ there is some evidence for a bidirectional biological interaction between internalising disorders and gastrointestinal conditions.^1566^,^68^ While an intestinal bacteria imbalance affects one’s mood, this imbalance by itself could be caused by stress, anxiety and depression.^66^,^68^

The adolescents in class 4 were more often female, older, had less-educated parents and had one of the highest rates of healthcare use and costs and youth care use. Other studies have confirmed this increased healthcare use among adolescents with psychosomatic conditions and internalising disorders.^64^ Adolescents with internalising behavioural disorders and multimorbidity were more often in contact with family protection services and were likelier to be registered as having dropped out of school and as being victims of a criminal act. In short, in this group of adolescents, social and family complexities seem to drive the clustering of (mental) health problems. Conversely, psychosomatic conditions may increase the risk of social problems, as internalising disorders were found to increase the risk of school dropout.^64^ The combination of both health and social problems in our data calls for approaches that go beyond treatments targeting individual issues. Consequently, we recommend that healthcare shift towards recognising the diverse perspectives and needs of young patients and strengthening interdisciplinary collaboration between healthcare providers and social support systems.

### Strengths and limitations

To the best of our knowledge, this is the first study to explore multimorbidity patterns in adolescents using GP-registered data combined with societal data. This data combination allowed us to include a wide range of ICPC codes, healthcare use and costs, and social contextual factors on the individual, household and parental levels over a longer time period. In addition, using GP-registered health data eliminated the risk of information and recall bias by individuals. Further, in the study of Nichols *et al.,*^39^ LCA was found to be superior to other clustering techniques in identifying multimorbidity classes in a simulated dataset where the actual clusters were known.^39^ Finally, due to the sources of data we utilised the population included in our study is representative for adolescents living in Dutch cities, as it consisted of a large adolescent primary care population with significant differences in health and social well-being.

Our study does have some limitations. First, by using GP registration data, we could not account for potential GPs implicit bias, undiagnosed, incorrectly diagnosed or unreported health conditions, and we cannot draw conclusions about the severity of the registered health conditions. Although we discussed our data with GPs, implicit bias remains an important issue, as it may affect diagnostic and treatment decisions and warrants targeted training. Additionally, the actual number of adolescents with multimorbidity may be higher, as adolescents may not always visit the GP while having symptoms, or may visit a GP outside their own neighbourhood or city. Also, not all GPs in The Hague and Leiden participate in ELAN, so their patients were not captured in our dataset. Second, complete information on social contextual factors was not available for all adolescents; in some cases, one or both parents were not registered in SN or ELAN or their registered information was incomplete, missing factors such as education level. Although information on the country of birth of the individuals and their parents was included, data on immigration status and the duration of residence of families in the Netherlands were not available. Third was that, because we analysed a 2-year period, we did not know if health conditions were present at the same time, and we were not able to identify causal relationships. Similarly, as we did not investigate the interactions between clustered health conditions, we were only able to identify likely syndemic vulnerability rather than definitively determine the presence of a syndemic. Finally, although LCA is an interpretable method to group individuals and the four-class model demonstrated a reliable classification precision with an entropy of 0.91, a limitation arises in assigning adolescents to a class based solely on the largest posterior probability, as not all adolescents fully align with a single class. This should be considered when interpreting the results.

## Conclusions

One quarter of adolescents aged 10–19 year-old, registered at primary care practices in the Dutch cities The Hague and Leiden, experienced multimorbidity between 2018 and 2019. Within this group, four multimorbidity classes were identified, shaped by social contextual factors on the individual, household and parental levels. Adolescents in classes 3 and 4, characterised by externalising or internalising disorders with physical and somatic health conditions, most frequently exhibited adverse social contextual factors, such as school dropout. Increased healthcare use and costs across all classes indicate a higher disease burden for adolescents with clustered health conditions compared with those with a single or no condition. This shows the importance of considering the interaction and co-occurrence of health conditions in diagnosis and treatment, and the collaboration with social services.

Our findings highlight the importance of implementing integrated preventative strategies addressing multilevel aspects to reduce potential syndemic vulnerability. The perspectives and needs of young patients and their families should be prioritised, and interdisciplinary collaboration between healthcare providers and social support systems should be promoted. We recommend validating the current LCA model in a comparable study population to clarify whether the identified classes are present in other adolescent primary care population groups. Furthermore, future studies should focus on a longitudinal approach to identify the causal relationships among clustered health conditions and social contextual factors across the life course. Monitoring adolescents’ syndemic vulnerability over time will provide a deeper understanding of health and social inequalities later in life.

## Supplementary material

10.1136/bmjph-2024-002032 online supplemental file 1

10.1136/bmjph-2024-002032 online supplemental file 2

10.1136/bmjph-2024-002032 online supplemental file 3

## Data Availability

No data are available.

## References

[1] van den Akker M, Dieckelmann M, Hussain MA (2022). Children and adolescents are not small adults: toward a better understanding of multimorbidity in younger populations. J Clin Epidemiol.

[2] Armocida B, Monasta L, Sawyer S (2022). Burden of non-communicable diseases among adolescents aged 10-24 years in the EU, 1990-2019: a systematic analysis of the Global Burden of Diseases Study 2019. Lancet Child Adolesc Health.

[3] Sawyer SM, Afifi RA, Bearinger LH (2012). Adolescence: a foundation for future health. Lancet.

[4] Minicucci C, National Academies of Sciences E, Medicine, Health, Medicine D, Board on Global H, Forum on Microbial T Using syndemic theory and the societal lens to inform resilient recovery from COVID-19: Toward a post-pandemic World: proceedings of a workshop—in brief.

[5] Slagboom MN, Crone MR, Reis R (2022). Exploring syndemic vulnerability across generations: A case study of a former fishing village in the Netherlands. Soc Sci Med.

[6] Tsai AC, Mendenhall E, Trostle JA (2017). Co-occurring epidemics, syndemics, and population health. Lancet.

[7] Mendenhall E, Newfield T, Tsai AC (2022). Syndemic theory, methods, and data. Soc Sci Med.

[8] Mendenhall E, Kohrt BA, Logie CH (2022). Syndemics and clinical science. Nat Med.

[9] Slagboom MN, Reis R, Tsai AC (2021). Psychological distress, cardiometabolic diseases and musculoskeletal pain: A cross-sectional, population-based study of syndemic ill health in a Dutch fishing village. J Glob Health.

[10] Singer M (1996). A dose of drugs, a touch of violence, a case of AIDS: Conceptualizing the SAVA syndemic. Free Inq Creat Sociol.

[11] Barnett K, Mercer SW, Norbury M (2012). Epidemiology of multimorbidity and implications for health care, research, and medical education: a cross-sectional study. Lancet.

[12] Merikangas KR, Nakamura EF, Kessler RC (2009). Epidemiology of mental disorders in children and adolescents. Dialogues Clin Neurosci.

[13] Ferro MA, Lipman EL, Van Lieshout RJ (2019). Multimorbidity in Children and Youth Across the Life-course (MY LIFE): protocol of a Canadian prospective study. BMJ Open.

[14] Romano I, Buchan C, Baiocco-Romano L (2021). Physical-mental multimorbidity in children and youth: a scoping review. BMJ Open.

[15] Tegethoff M, Belardi A, Stalujanis E (2015). Association between mental disorders and physical diseases in adolescents from a nationally representative cohort. Psychosom Med.

[16] Tang J, Ma Y, Hoogendijk EO (2024). Associations between healthy lifestyle and mortality across different social environments: a study among adults with frailty from the UK Biobank. Eur J Public Health.

[17] Nicolson KP, Mills SEE, Senaratne DNS (2023). What is the association between childhood adversity and subsequent chronic pain in adulthood? A systematic review. *BJA Open*.

[18] Berger E, Castagné R, Chadeau-Hyam M (2019). Multi-cohort study identifies social determinants of systemic inflammation over the life course. Nat Commun.

[19] Bencsik P, Halliday TJ, Mazumder B (2023). The intergenerational transmission of mental and physical health in the United Kingdom. J Health Econ.

[20] Larsen FB, Pedersen MH, Friis K (2017). A Latent Class Analysis of Multimorbidity and the Relationship to Socio-Demographic Factors and Health-Related Quality of Life. A National Population-Based Study of 162,283 Danish Adults. PLoS One.

[21] Strozza C, Pasqualetti P, Egidi V (2020). Health profiles and socioeconomic characteristics of nonagenarians residing in Mugello, a rural area in Tuscany (Italy). BMC Geriatr.

[22] Olaya B, Moneta MV, Caballero FF (2017). Latent class analysis of multimorbidity patterns and associated outcomes in Spanish older adults: a prospective cohort study. BMC Geriatr.

[23] Park B, Lee HA, Park H (2019). Use of latent class analysis to identify multimorbidity patterns and associated factors in Korean adults aged 50 years and older. PLoS One.

[24] Roomaney RA, van Wyk B, Cois A (2022). Multimorbidity patterns in South Africa: A latent class analysis. Front Public Health.

[25] Lindley LC, Mack JW, Bruce DJ (2016). Clusters of Multiple Complex Chronic Conditions: A Latent Class Analysis of Children at End of Life. J Pain Symptom Manage.

[26] Carrilero N, Dalmau-Bueno A, García-Altés A (2020). Comorbidity patterns and socioeconomic inequalities in children under 15 with medical complexity: a population-based study. BMC Pediatr.

[27] Hesketh KR, Fagg J, Muniz-Terrera G (2016). Co-occurrence and clustering of health conditions at age 11: cross-sectional findings from the Millennium Cohort Study. BMJ Open.

[28] Voisin DR, Takahashi LM (2021). The Relationship Between Violence Syndemics and Sexual Risk Behaviors Among African American Adolescents: Implications for Future Research. J Adolesc Health.

[29] Okumu M, Ombayo BK, Small E (2019). Psychosocial Syndemics and Sexual Risk Practices Among U.S. Adolescents: Findings from the 2017 U.S. Youth Behavioral Survey. Int J Behav Med.

[30] Choi AY, Israel T, Nylund-Gibson K (2020). Syndemic behavioral risk and suicidality among bisexual adolescents: A latent class analysis. J Consult Clin Psychol.

[31] Lett E, Abrams MP, Moberg E (2022). Syndemic relationship of depressive symptoms, substance use, and suicidality in transgender youth: a cross-sectional study using the U.S. youth risk behavior surveillance system. Soc Psychiatry Psychiatr Epidemiol.

[32] Pons-Vigués M, Diez È, Morrison J (2014). Social and health policies or interventions to tackle health inequalities in European cities: a scoping review. BMC Public Health.

[33] Ardesch FH, Meulendijk MC, Kist JM (2023). The introduction of a data-driven population health management approach in the Netherlands since 2019: The Extramural LUMC Academic Network data infrastructure. Health Policy.

[34] Bakker BFM, van Rooijen J, van Toor L (2014). The System of social statistical datasets of Statistics Netherlands: An integral approach to the production of register-based social statistics. Statistical Journal of the IAOS.

[35] Statistieken gemeente den haag allecijfers. https://allecijfers.nl/gemeente/den-haag.

[36] Statistieken gemeente leiden: allecijfers. https://allecijfers.nl/gemeente/leiden.

[37] Sociaal-economische status; scores per wijk en buurt, regio-indeling 2021. https://www.cbs.nl/nl-nl/cijfers/detail/85163NED.

[38] Cornish RP, Boyd A, Van Staa T (2013). Socio-economic position and childhood multimorbidity: a study using linkage between the Avon Longitudinal Study of Parents and Children and the General Practice Research Database. Int J Equity Health.

[39] Nichols L, Taverner T, Crowe F (2022). In simulated data and health records, latent class analysis was the optimum multimorbidity clustering algorithm. J Clin Epidemiol.

[40] Weller BE, Bowen NK, Faubert SJ (2020). Latent Class Analysis: A Guide to Best Practice. Journal of Black Psychology.

[41] Laugesen B, Mohr-Jensen C, Boldsen SK (2018). Attention Deficit Hyperactivity Disorder in Childhood: Healthcare Use in a Danish Birth Cohort during the First 12 Years of Life. J Pediatr.

[42] Vera-Toscano E, Brown H (2021). The Intergenerational Transmission of Mental and Physical Health in Australia: Evidence Using Data From the Household Income and Labor Dynamics of Australia Survey. Front Public Health.

[43] Kaplan SH, Shaughnessy M, Fortier MA (2022). The role of parental health and distress in assessing children’s health status. Qual Life Res.

[44] Berkelbach van der Sprenkel EE, Nijhof SL, Dalmeijer GW (2022). Psychosocial functioning in adolescents growing up with chronic disease: The Dutch HBSC study. Eur J Pediatr.

[45] Göbel K, Cohrdes C (2021). The whole is greater than the sum of its parts: profiles of multiple mental health risk factors using Latent class analysis. Child Adolesc Psychiatry Ment Health.

[46] Paiva V, Ayres JR de CM, França Junior I (2020). From “combined prevention” to “comprehensive prevention”: building the response to the syndemic with adolescents and youth in São Paulo, Brazil (2020-2023). Cad Saúde Pública.

[47] Cheng TL, Johnson SB, Goodman E (2016). Breaking the Intergenerational Cycle of Disadvantage: The Three Generation Approach. Pediatrics.

[48] Groenestein SFF, Crone MR, Dubbeldeman EM (2025). Exploring Family Typologies and Health Outcomes in a Dutch Primary Care Population of Children Living in Urban Cities in the Netherlands: A Latent Class Analysis. Int J Environ Res Public Health.

[49] Nandyala A, Dougherty C (2022). Dermatologic Symptoms and Syndromes Associated with Headache. Curr Pain Headache Rep.

[50] Silverberg JI (2016). Association between childhood eczema and headaches: An analysis of 19 US population-based studies. J Allergy Clin Immunol.

[51] Paller AS, Spergel JM, Mina-Osorio P (2019). The atopic march and atopic multimorbidity: Many trajectories, many pathways. J Allergy Clin Immunol.

[52] Yaneva M, Darlenski R (2021). The link between atopic dermatitis and asthma- immunological imbalance and beyond. Asthma Res and Pract.

[53] Hu C, Nijsten T, Pasmans S (2020). Associations of eczema phenotypes with emotional and behavioural problems from birth until school age. The Generation R Study. *Br J Dermatol*.

[54] Keller W, Vogel M, Prenzel F (2021). Atopic diseases in children and adolescents are associated with behavioural difficulties. BMC Pediatr.

[55] Mogensen N, Larsson H, Lundholm C (2011). Association between childhood asthma and ADHD symptoms in adolescence--a prospective population-based twin study. Allergy.

[56] Chen M-H, Su T-P, Chen Y-S (2013). Asthma and attention-deficit/hyperactivity disorder: a nationwide population-based prospective cohort study. J Child Psychol Psychiatry.

[57] Elenkov IJ (2004). Glucocorticoids and the Th1/Th2 balance. Ann N Y Acad Sci.

[58] Buske-Kirschbaum A, Trikojat K, Tesch F (2019). Altered hypothalamus-pituitary-adrenal axis function: A relevant factor in the comorbidity of atopic eczema and attention deficit/hyperactivity disorder?. Psychoneuroendocrinology.

[59] Bevaart F, Mieloo CL, Jansen W (2012). Ethnic differences in problem perception and perceived need for care for young children with problem behaviour. *J Child Psychol Psychiatry*.

[60] Oude Groeniger J, Houweling TA, Jansen PW (2023). Social inequalities in child development: the role of differential exposure and susceptibility to stressful family conditions. J Epidemiol Community Health.

[61] Rodriguez G, Hartley SL, Bolt D (2019). Transactional Relations Between Parenting Stress and Child Autism Symptoms and Behavior Problems. J Autism Dev Disord.

[62] Lawrence KC, Adebowale TA (2023). Adolescence dropout risk predictors: Family structure, mental health, and self-esteem. J Community Psychol.

[63] Saladino V, Mosca O, Petruccelli F (2021). The Vicious Cycle: Problematic Family Relations, Substance Abuse, and Crime in Adolescence: A Narrative Review. Front Psychol.

[64] Hetlevik Ø, Bøe T, Hysing M (2018). GP-diagnosed internalizing and externalizing problems and dropout from secondary school: a cross-sectional study. Eur J Public Health.

[65] Yacob D, Di Lorenzo C, Bridge JA (2013). Prevalence of pain-predominant functional gastrointestinal disorders and somatic symptoms in patients with anxiety or depressive disorders. J Pediatr.

[66] Dinan TG, Cryan JF (2017). The Microbiome-Gut-Brain Axis in Health and Disease. Gastroenterol Clin North Am.

[67] Bisgaard TH, Allin KH, Keefer L (2022). Depression and anxiety in inflammatory bowel disease: epidemiology, mechanisms and treatment. *Nat Rev Gastroenterol Hepatol*.

[68] Koloski NA, Jones M, Kalantar J (2012). The brain–gut pathway in functional gastrointestinal disorders is bidirectional: a 12-year prospective population-based study. Gut.

