# Supplementary material for: Exploring syndemic vulnerability among adolescents living in urban cities in the Netherlands: a latent class analysis
Source: BMJ Public Health. 2026 Jan 19;4(1):e002032. doi: 10.1136/bmjph-2024-002032 (PMC12820875; doi:10.1136/bmjph-2024-002032)
Supplement: online supplemental file 1 [file bmjph-4-1-s001.docx]

# Appendix

## **Appendix 1.** Overview of health conditions and their ICPC codes.

| Health condition category | ICPC* | Additional Information |
| --- | --- | --- |
| Endocrine disorders | T90, T90.01, T90.02, L88, L88.01, L88.02 | Including diabetes, rheumatoid arthritis and thyroid conditions |
| Cancer | A79, B72, B72.01, B72.02, B73, D75, D76, D77, D77.01, D77.02, D77.03, D77.04, L71, L71.01, L71.01, N74, R84, R85, S77, T71, U75, U76, U77, W72, S77.02, S77.03, S77.04, X75, X76, X76.01, X77, X77.01, X77.02, Y78.01, Y78.02, Y78.03, Y77, Y78 |  |
| Cardiac disease | K78, K79.01, K79.02, K80.01, K80.02, K80.03, K79, K80,  K74, K74.01, K74.02, K76.01, K76.02, K75, K76, K86, K87 | Including heart rhythm disorders, coronary heart diseases, and hypertensive heart diseases |
| (Chronic) Neck and back conditions | L01, L02, L03, L83, L83.01, L84, L84.01, L84.02, L86.01, L86 |  |
| Headache disorders | N01, N02, N90, N89 | Including migraine |
| Asthma | R96, R96.01, R96.02 |  |
| Gastrointestinal conditions | D93,D94, D94.01, D94.01, D85, D86, D86.01, D88-D91, D91.01, D91.02, D91.03, D98, D98.01, D98.02, D98.03, D01, D02, D04, D06, Y02 | Including irritable bowel syndrome, colitis ulcerosa, digestive ulcers, acute abdominal conditions, and other gastrointestinal conditions or pain complaints |
| Generalized fatigue/pain | A01, A04, A04.01 |  |
| Skin conditions | S86, S86.01, S86.02, S88.01, S88.02, S88.03, S88.04, S87, S88, S89, S90, S91 | Including eczema and psoriasis |
| Overweight/Obesity | T82, T83 |  |
| (Head) Trauma and fractures | A80, A81, A82, N79, N80, N80.01, N80.02, N80.03, N80.04, N81 |  |
| Epilepsy | N88 |  |
| Sexual behavior | W82, W83, X70, X71, X73, X74, X74.01, B90.01, B90.02, X90, X91, Y70, Y71, Y72, Y76, B90 | Including abortion, miscarriage, and sexually transmitted diseases |
| Externalizing behavioral disorders | P21, P22, P23, P04 | Includes symptoms of anger/behavioral issues and ADHD |
| Internalizing disorders | P03, P76, P76.01, P76.02, P73.02, P73, P74, P79, P79.01, P79.02, P74.01, P74.02, P02.01, P01 | Including bipolar disorder, symptoms of feeling down or depressed, posttraumatic stress disorder, and feelings of anxiety |
| Burnout/Stress | P78, P02, P06, P06.01, P07 | Including burnout, sleeping disorders, and stress responses |
| Suicide attempts | P77, P77.01, P77.02 |  |
| Substance abuse | P15, P15.01, P15.02, P15.03, P15.05, P15.06, P19.01, P19.02, P16, P17, P18, P19 | Including alcohol, tobacco, medicines, and drugs |
| Other mental disorders | P72, P98, P80, P80.01, P80.02, P99.01,T06, T06.01, T06.02,P99.02, P99, P78 | Including schizophrenia, autism spectrum disorders, personality disorders, eating disorders, adjustment disorders, and undefined mental disorders |
| * In the Netherlands, GPs register symptoms and diagnoses of health conditions as episodes using the ICPC [1]. The presence of the included health conditions was established through the ICPC codes belonging to both symptoms and diagnosis. Classification of ICPC codes was based on the classification of Oostrom et al., and by knowledge of the researchers [2]. | | |

## **Appendix 2. Overview of Sociodemographic and Social Contextual Factors.**

| Factor | Definition and additional background information | Categories | Data source* |
| --- | --- | --- | --- |
| Individual level |  |  |  |
| Age | Age of adolescents in years calculated on 01-01-2018. | 10-14 years old  15-19 years old | SSD |
| Sex | Sex of the individual as stated at birth. | Male  Female | SSD |
| Country of origin | Country of origin based on the individual's birthplace and the birthplace of the individual's parents. An individual is categorized as "Dutch" if both the person and their parents were born in the Netherlands. If the mother’s country of birth differs from the Netherlands, this country determines the classification. If the father was born in a country other than the Netherlands but the mother was born in the Netherlands, then the father’s country of birth determines the classification. If both parents were born outside the Netherlands, the mother’s country of birth is used as the determining factor. Categorized based on the division of SSD and adjusted by the authors to fit the characteristics of the study population. The Hague and Leiden are considered international cities with many ethnicities. Categories are based on frequent countries of origin in the Netherlands. Surinam, Indonesia and Dutch-Caribbean are combined based on the historical background of Dutch colonization in these countries. | The Netherlands  Europe (excluding the Netherlands)  Turkey/Morocco  Surinam/ Indonesia/ Dutch-Caribbean  Other (Africa/Asia/America/Oceania) | SSD |
| Juvenile Crime Suspect | An individual is considered a juvenile crime suspect if they have been sent to HALT or the Dutch Juvenile Probation Services. HALT is an organization in the Netherlands focusing on preventing and punishing juvenile crime. HALT defines juvenile crime as the behavior of young people aged 12-23 that violate certain norms laid down in law. An individual is known to the Dutch Juvenile Probation Services if they have been convicted of a crime.  Included years: 2015-2019. | No  Yes | SSD |
| School dropout | Leaving (high) school prematurely without obtaining a degree.  Included years: 2015-2019. | No  Yes | SSD |
| Victim of a criminal act | Registered victim of any criminal act reported to the police. The criminal acts may vary from theft of belongings to sexual assault.  Included years: 2015-2019. | No  Yes | SSD |
| Social problems | Family, partner, and other social problems as registered by the GP.  Included years: 2018-2019.  Included ICPC codes:  *Z04, Z04.01, Z04.02, Z04.03, Z04.04, Z04.05, Z24, Z27, Z28, Z29 Z29.01, Z29.02, Z29.03*  *Z16, Z16.01, Z16.02, Z16.03, Z18, Z19, Z20, Z21, Z21.01, Z21.02, Z22, Z23*  *Z12, Z12.01, Z12.02, Z13, Z13.01, Z13.02, Z13.03, Z14, Z15* | No  Yes | ELAN |
| Household level |  |  |  |
| Single parent household | Registered single-parent household in 2018. Living in a two-parent household does not necessarily consist of the individual's biological parents. An individual is considered living in a two-parent household if the household type registered includes two adults, married or unmarried, with children. | No  Yes | SSD |
| Household composition | Number of persons registered at the same home address in 2018. | 4 or less  More than 4 | SSD |
| Known at Youth Protection Services | Received services and support through the Dutch Youth Protection Services. Adolescents are known by the Dutch Youth Protection Services if their family environment is unsafe. The juvenile court decides on a child protection measure to eliminate any threats to the safe development of a child. This decision follows an investigation by the Council for Child Protection.  Included years: 2015-2019. | No  Yes | SSD |
| Household income | The household income is based on percentage groups of standardized disposable income of private households;  <10^th^ percentile is considered low, 10^th^ – 90^th^ percentile is considered moderate, and > 90^th^ percentile is considered high. 'Other' includes individuals with an unknown household income or belonging to a student or institutionalized household.  Household income for the year 2018 was used to determine the income category of each individual at the start of 2018. | Low  Moderate  High  Other | SSD |
| Socioeconomic Status  neighborhood | Mean SES of the neighborhood one lives in, measured by the SES-WOA score by CBS in 2019 [3]. | Low  Average  High | SSD |
| Parental level |  |  |  |
| Parent(s) having debts** | Based on registration of debt restructuring and/or delayed health insurance payments for more than six months for one or both parents.  Included years: 2015-2019. | No  Yes | SSD |
| Parent(s) being detained or suspect of a crime** | One or both parents being detained or suspect of a crime.  Included years: 2015-2019. | No  Yes | SSD |
| Parent(s) being victim of a criminal act ** | Registered victim of any criminal act reported to the police of one or both parents. The criminal acts may vary from theft of belongings to sexual assault.  Included years: 2015-2019. | No  Yes | SSD |
| Parent(s) being divorced** | One or both parents have been divorced or there was a shift from a two-parent household to a single-parent household.  Included years: 2015-2019. | No  Yes | SSD |
| Highest achieved educational level parent(s)** | Highest completed education, according to the International Standard Classification of Education and following categorization of Statistics Netherlands. Highest achieved educational level in the family, from either the mother or the father. Low included primary school and junior high school. Middle included senior high school and MBO. High included HBO and WO.  Included years: 2015-2018. | Low  Middle  High  Missing | SSD |
| Parent(s) with a chronic physical health condition*** | One or both parents with a chronic physical condition between 2016 and 2019.  The (chronic) health conditions are:  *Diabetes, Cancer, Heart Failure, Coronary Heart Disease, Stroke, Rheumatism, Migraine, Chronic Obstructive Pulmonary Disease (COPD), Asthma, Dementia, Hypertensive Heart Disease, Colitis Ulcerosa, (Head) Trauma, Thyroid Disorder, Stomach Ulcer, Sexually Transmitted Infections/HIV, Eczema, Osteoporosis, Miscarriage, Abortion, Osteoarthritis, Parkinson’s Disease, Epilepsy.* | None  One parent  Both parents | ELAN |
| Parent(s) with a mental disorder*** | One or both parents with mental health conditions between 2016 and 2019.  The mental health conditions are:  *Suicide attempts, Burnout, Mood Disorder, Anxiety Disorder, Substance Abuse, Personality Disorder, Psychotic Disorder, Attention Deficit Hyperactivity Disorder (ADHD), Autism Spectrum Disorder (ASD), Eating Disorder.* | None  One parent  Both parents | ELAN |
| Parent(s) with a somatic health  condition*** | One or both parent with a somatic health condition between 2016 and 2019.  The included health conditions are:  *Headache disorders, Generalized fatigue/pain, Irritable bowel syndrome (IBS), Abdominal pain, (Chronic) Neck and back Pain* | None  One parent  Both parents | ELAN |
| Parent(s) with social problems | Family, partner, and other social problems as registered by the GP.  Included ICPC codes:  *Z04, Z04.01, Z04.02, Z04.03, Z04.04, Z04.05, Z24, Z27, Z28, Z29 Z29.01, Z29.02, Z29.03*  *Z16, Z16.01, Z16.02, Z16.03, Z18, Z19, Z20, Z21, Z21.01, Z21.02, Z22, Z23*  *Z12, Z12.01, Z12.02, Z13, Z13.01, Z13.02, Z13.03, Z14, Z15.* | No  Yes | ELAN |
| Healthcare use and costs |  |  |  |
| GP healthcare use | Total number of GP visits during the included time period 2018-2019. Excluding repeat prescriptions, flu vaccines, internal consultations, mail processing, notes and missed appointments.  The number of annual GP visits was categorized based on the percentiles observed in the multimorbidity group. | No visits  1-3 visits  4-6 visits  More than 6 visits | ELAN |
| Healthcare costs | Includes all costs in euros associated with the Health Insurance Act (e.g. GP costs and hospital care costs), excluding youth care costs.  Mean healthcare costs between 2018 and 2019.  Categorization was based on the lowest possible maximum personal contribution toward healthcare costs in the Netherlands, which is 385 euros per year for individuals aged 18 years and older. | 0-385  385-2000  2000 or more | SSD |
| In contact with youth care | Children to whom, during (part of) the reporting periods, one or more forms of  assistance or care were provided under the Youth Act, such as support for psychological or parenting problems, excluding youth protection services  and youth rehabilitation  Included years: 2015-2019. | No  Yes | SSD |
| *Data sources used in the current study consist of registered primary care records of general practices connected to the Extramural Leiden Academic Network (ELAN) primary care network was linked to routinely collected societal data of Statistics Netherlands (SN) [4].  ** Information of parents was linked to individuals through a unique parent-child identification code. Data on the parents is missing if no parent could be linked to the adolescent through the parent-child linking code. This may suggest that the parent is not registered in SSD (not a resident in the Netherlands) or as the individual's parent.  *** To attain Information on the parent's health status, the adolescents were linked through the unique parent-child identification code. Missing Information on the parent's health can result from no parent being linked to the adolescent (see missing Information above) in the SSD environment. Information is also missing if the parent is not registered at a general practice connected to ELAN. | | | |

# References

1. Duineveld B, Kole, H.M., van Werven, H. Richtlijn adequate dossiervorming met het elektronisch patiëntdossier. 2019.

2. Oostrom SH, van Gijsen, R., Stirbu, I., Korevaar, J.C., Schellevis, F.G., Picavet, H.S.J., Hoeymans, N. Toename in chronische ziekten en multimorbiditeit: veroudering van de bevolking verklaart maar een deel van de toename. Nederlands Tijdschrift voor Geneeskunde. 2017;161(34):D1429.

3. CBS. Sociaal-economische status; scores per wijk en buurt, regio-indeling 2021 2022. Available from: https://opendata.cbs.nl/#/CBS/nl/dataset/85163NED/table

4. Ardesch FH, Meulendijk MC, Kist JM, Vos RC, Vos HMM, Kiefte-de Jong JC, et al. The introduction of a data-driven population health management approach in the Netherlands since 2019: The Extramural LUMC Academic Network data infrastructure. Health Policy. 2023;132:104769.
