# Supplementary material for: Exploring syndemic vulnerability among adolescents living in urban cities in the Netherlands: a latent class analysis
Source: BMJ Public Health. 2026 Jan 19;4(1):e002032. doi: 10.1136/bmjph-2024-002032 (PMC12820875; doi:10.1136/bmjph-2024-002032)
Supplement: online supplemental file 2 [file bmjph-4-1-s002.docx]

**Supplementary Figure 1. Plot of health conditions by the four-multimorbidity classes identified by Latent Class Analysis**


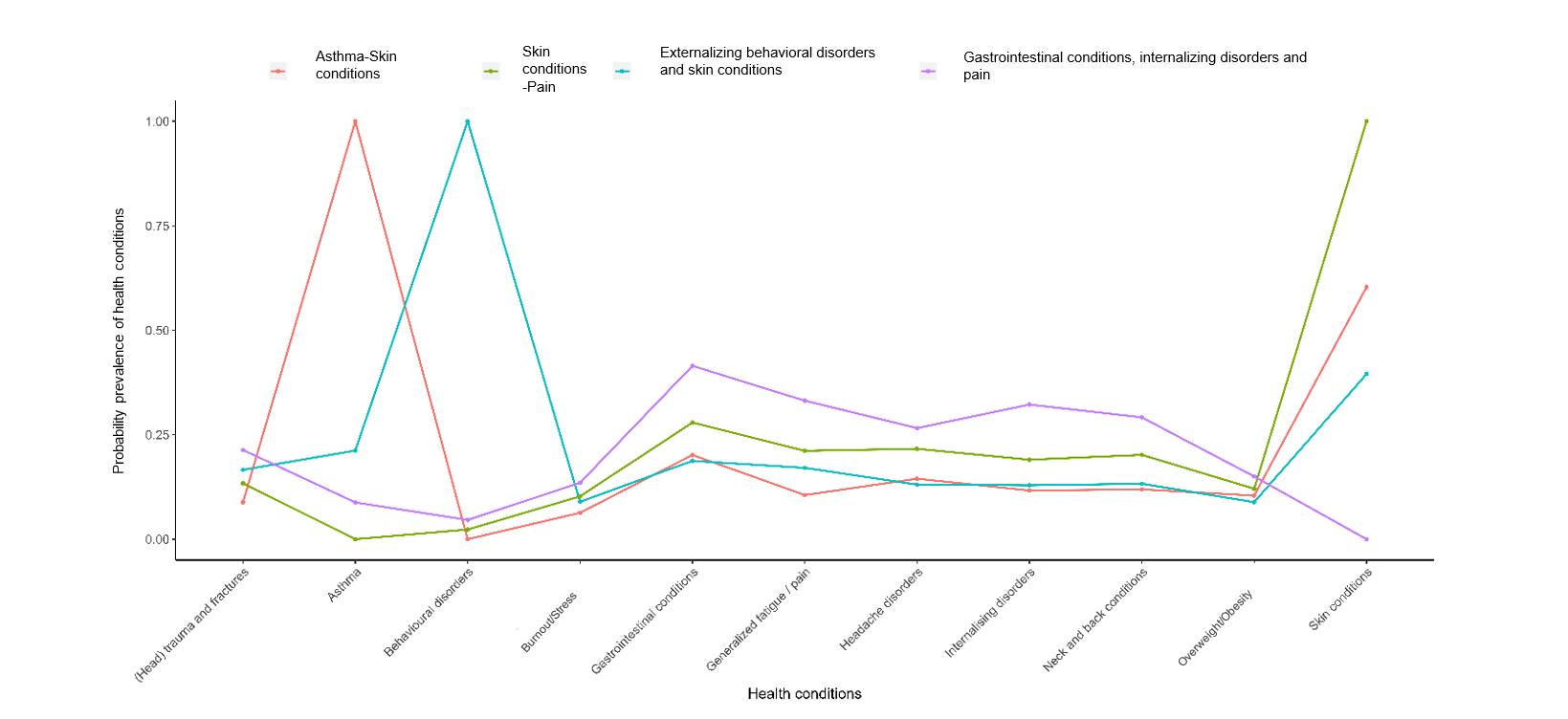


Prevalence of the health conditions is displayed on the vertical axis (1.00 means probability prevalence of 100% within the class). Probability prevalence is derived from the posterior probabilities of an individual in the class having the condition.
