# Supplementary material for: Exploring syndemic vulnerability among adolescents living in urban cities in the Netherlands: a latent class analysis
Source: BMJ Public Health. 2026 Jan 19;4(1):e002032. doi: 10.1136/bmjph-2024-002032 (PMC12820875; doi:10.1136/bmjph-2024-002032)
Supplement: online supplemental file 3 [file bmjph-4-1-s003.docx]

| Supplementary Table 1. Model fit statistics of the Latent Class Analysis models. | | | | | | |
| --- | --- | --- | --- | --- | --- | --- |
| ^1^Model | **Akaike information criterion (AIC)^2^** | **Bayesian Information Criterion (BIC)^2^** | **G-Square** | **Chi-square goodness of fit** | **Residual degrees of freedom** | **Log Likelihood** |
| 1 | 31122.79 | 31188.03 | 2522.844 | 3425.025 | 2036 | -15550.39 |
| 2 | 30916.76 | 31053.16 | 2292.814 | 5508.484 | 2024 | -15435.38 |
| 3 | 30801.6 | 31009.17 | 2153.65 | 12922.33 | 2012 | -15365.8 |
| 4 | **30690.79** | **30969.52** | **2018.841** | **12462.28** | **2000** | **-15298.39** |
| 5 | 30653.68 | 31003.59 | 1957.738 | 8046.625 | 1988 | -15267.84 |
| 6 | 30568 | 30989.07 | 1848.058 | 7465.954 | 1976 | -15213 |
|  |  |  |  |  |  |  |
| ^1^The model represents the number of classes included in the LCA model. Model 4 represents a model with four latent classes. ^2^ Models up to six latent classes were executed using a stepwise approach, which allows for the determination of a specific number of groups, after which we assessed whether this number was sufficient. Maximum iterations were set at 8,000 and random starting values were set at 15. We assessed model fit based on fit statistics (i.e., the Bayesian information criterion (BIC) and the Akaike information criterion (AIC)) and the interpretability of the latent classes. Lower BIC and AIC values indicated a better-fitting model. Additionally, we assessed the models’ entropy, indicating the clarity of the profile distinction, with scores ranging from 0–1 (optimal) [40]. | | | | | | |

| Supplementary table 2: Characteristics of social contextual factors and multimorbidity classes | | | | | | |
| --- | --- | --- | --- | --- | --- | --- |
|  | **No morbidity** | **Single morbidity** | **Class 1** | **Class 2** | **Class 3** | **Class 4** |
|  | n=4780 (44.1%) | n=3.280 (30.3%) | n=442  (4.1%)^1^ | n= 794  (7.3%)^1^ | n= 565 (5.2%)^1^ | n=980  (9.0%)^1^ |
|  | n(%) | n(%) | n(%) | n(%) | n(%) | n(%) |
| Age ** |  |  |  |  |  |  |
| 10-14 years | 2797 (58.5) | 1795 (54.7) | 230 (52.0) | 415 (52.3) | 321 (56.8) | 396 (40.4) |
| 15-19 years | 1983 (41.5) | 1485 (45.3) | 212 (48.0) | 379 (47.7) | 244 (43.2) | 584 (59.6) |
| Sex ** |  |  |  |  |  |  |
| Male | 2600 (54.4) | 1601 (48.8) | 238 (53.8) | 316 (39.8) | 351 (62.1) | 369 (37.7) |
| Female | 2180 (45.6) | 1679 (51.2) | 204 (46.2) | 478 (60.2) | 214 (37.9) | 611 (62.3) |
| Country of origin ** |  |  |  |  |  |  |
| The Netherlands | 2113 (44.2) | 1503 (45.8) | 185 (41.9) | 345 (43.5) | 305 (54.0) | 433 (44.2) |
| Europe (excl. The Netherlands | 427 (8.9) | 240 (7.3) | 25 (5.7) | 57 (7.2) | 37 (6.5) | 73 (7.4) |
| Turkey/Morocco | 971 (20.3) | 765 (23.3) | 119 (26.9) | 200 (25.2) | 85 (15.0) | 249 (25.4) |
| Suriname/Indonesia/Dutch- Caribbean | 514 (10.8) | 352 (10.7) | 45 (10.2) | 95 (12.0) | 66 (11.7) | 108 (11.0) |
| Other(Africa/Asia/America/Oceania) | 755 (15.8) | 420 (12.8) | 68 (15.4) | 97 (12.2) | 72 (12.7) | 117 (11.9) |
| Juvenile Crime Suspect ** |  |  |  |  |  |  |
| No | 4633 (96.9) | 3147 (95.9) | 423 (95.7) | 772 (97.2) | 525 (92.9) | 929 (94.8) |
| Yes | 147 (3.1) | 133 (4.1) | 19 (4.3) | 22 (2.8) | 40 (7.1) | 51 (5.2) |
| School dropout ** |  |  |  |  |  |  |
| No | 4588 (96.0) | 3126 (95.3) | 416 (94.1) | 755 (95.1) | 512 (90.6) | 886 (90.4) |
| Yes | 192 (4.0) | 154 (4.7) | 26 (5.9) | 39 (4.9) | 53 (9.4) | 94 (9.6) |
| Victim of a criminal act ** |  |  |  |  |  |  |
| No | 4491 (94.0) | 3007 (91.7) | 412 (93.2) | 709 (89.3) | 483 (85.5) | 855 (87.2) |
| Yes | 289 (6.0) | 273 (8.3) | 30 (6.8) | 85 (10.7) | 82 (14.5) | 125 (12.8) |
| Social problems ** |  |  |  |  |  |  |
| No | 4597 (96.2) | 3101 (94.5) | 411 (92.8) | 723 (91.1) | 523 (92.6) | 892 (91.0) |
| Yes | 183 (3.8) | 179 (5.5) | 31 (7.2) | 71 (8.9) | 42 (7.4) | 88 (9.0) |
| Single parent household ** |  |  |  |  |  |  |
| No | 3607 (75.5) | 2469 (75.3) | 319 (72.2) | 574 (72.2) | 369 (65.3) | 687 (70.1) |
| Yes | 1173 (24.5) | 811 (24.7) | 123 (27.8) | 220 (27.7) | 196 (34.7) | 293 (29.9) |
| Household composition ** |  |  |  |  |  |  |
| 4 or less | 2963 (62.0) | 2144 (65.4) | 290 (65.6) | 542 (68.3) | 423 (74.9) | 693 (70.7) |
| More than 4 | 1817 (38.0) | 1136 (34.6) | 152 (34.4) | 252 (31.7) | 142 (25.1) | 287 (29.3) |
| Known at Youth Protection Services ** |  |  |  |  |  |  |
| No | 4714 (98.6) | 3216 (98.0) | - | 777 (97.9) | 533 (94.3) | 930 (94.9) |
| Yes | 66 (1.4) | 64 (2.0) | - | 17 (2.1) | 32 (5.7) | 50 (5.1) |
| Household income ** |  |  |  |  |  |  |
| Low | 702 (14.7) | 445 (13.6) | 65 (14.7) | 136 (17.1) | 89 (15.8) | 146 (14.9) |
| Moderate | 3177 (66.5) | 2394 (73.0) | 325 (73.5) | 574 (72.3) | 404 (71.5) | 702 (71.6) |
| High | 786 (16.4) | 390 (11.9) | 41 (9.3) | 74 (9.3) | 51 (9.0) | 104 (10.6) |
| Other | 115 (2.4) | 51 (1.6) | 11 (2.5) | 10 (1.3) | 21 (3.7) | 28 (2.9) |
| SES neighborhood ** |  |  |  |  |  |  |
| Low | 2539 (53.1) | 1766 (53.8) | 246 (55.7) | 435 (54.8) | 263 (46.5) | 529 (54.0) |
| Average | 446 (9.3) | 248 (7.6) | 37 (8.4) | 51 (6.4) | 39 (6.9) | 65 (6.6) |
| High | 1794 (37.5) | 1266 (38.6) | 159 (36.0) | 308 (38.8) | 263 (46.5) | 386 (39.4) |
| Parent(s) having debts ** |  |  |  |  |  |  |
| No | 4323 (90.4) | 2958 (90.2) | 400 (90.5) | 709 (89.3) | 479 (84.8) | 850 (86.7) |
| Yes | 457 (9.6) | 322 (9.8) | 42 (9.5) | 85 (10.7) | 86 (15.2) | 130 (13.3) |
| Parent(s) being detained or suspect of a crime ** |  |  |  |  |  |  |
| No | 4421 (92.5) | 2993 (91.3) | 410 (92.8) | 724 (91.2) | 489 (86.5) | 878 (89.6) |
| Yes | 359 (7.5) | 287 (8.8) | 32 (7.2) | 10 (8.8) | 76 (13.5) | 102 (10.4) |
| Parent(s) being victim of a criminal act * | |  |  |  |  |  |
| No | 3867 (80.9) | 2599 (79.2) | 365 (82.6) | 635 (80.0) | 430 (76.1) | 756 (77.1) |
| Yes | 913 (19.1) | 681 (20.8) | 77 (17.4) | 159 (20.0) | 135 (23.9) | 224 (22.9) |
| Parent(s) being divorced |  |  |  |  |  |  |
| No | 4274 (89.4) | 2906 (88.6) | 403 (91.2) | 704 (88.7) | 503 (89.0) | 872 (89.0) |
| Yes | 506 (10.6) | 374 (11.4) | 39 (8.8) | 90 (11.3) | 62 (11.0) | 108 (11.0) |
| Highest achieved educational level parent(s) ** |  |  |  |  |  |  |
| Low | 1148 (24.0) | 858 (26.2) | 127 (28.7) | 236 (29.7) | 142 (25.1) | 313 (31.9) |
| Middle | 842 (17.6) | 676 (20.6) | 101 (22.9) | 178 (22.4) | 144 (25.5) | 206 (21.0) |
| High | 1985 (41.5) | 1263 (38.5) | 157 (35.5) | 261 (32.9) | 212 (37.5) | 319 (32.6) |
| Missing | 805 (16.8) | 483 (14.7) | 57 (12.9) | 119 (15.0) | 67 (11.9) | 142 (14.5) |
| Parent(s) with a chronic physical health condition ** |  |  |  |  |  |  |
| None | 1847 (38.6) | 847 (25.8) | 66 (14.9) | 131 (16.5) | 92 (16.3) | 184 (18.8) |
| One parent | 2181 (45.6) | 1611 (49.1) | 241 (54.5) | 383 (48.2) | 305 (54.0) | 512 (52.2) |
| Both parents | 752 (15.7) | 822 (25.1) | 135 (30.5) | 280 (35.3) | 168 (29.7) | 284 (29.0) |
| Parent(s) with a mental disorder ** |  |  |  |  |  |  |
| None | 2977 (62.3) | 1544 (47.1) | 182 (41.2) | 313 (39.4) | 174 (30.8) | 352 (35.9) |
| One parent | 1525 (31.9) | 1400 (42.7) | 193 (43.7) | 365 (46.0) | 281 (49.7) | 477 (48.7) |
| Both parents | 278 (5.8) | 336 (10.2) | 67 (15.2) | 116 (14.6) | 110 (19.5) | 151 (15.4) |
| Parent(s) with a somatic health condition ** |  |  |  |  |  |  |
| None | 2048 (42.8) | 998 (30.4) | 112 (25.3) | 194 (24.4) | 139 (24.6) | 241 (24.6) |
| One parent | 2090 (43.7) | 1649 (50.3) | 221 (50.0) | 415 (52.3) | 319 (56.5) | 510 (52.0) |
| Both parents | 642 (13.4) | 633 (19.3) | 109 (24.7) | 185 (23.3) | 107 (18.9) | 229 (23.4) |
| Parent(s) with social problems ** |  |  |  |  |  |  |
| No | 3937 (82.4) | 2490 (75.9) | 316 (71.5) | 542 (68.3) | 359 (63.5) | 707 (72.1) |
| Yes | 843 (17.6) | 790 (24.1) | 126 (28.5) | 252 (31.7) | 206 (36.5) | 273 (27.9) |
| *: p<0.05 for n(%)  **:p<0.001 for n(%)  Following the guidelines of Statistics Netherlands. cell counts below n=10 are not shown (-).  A detailed overview of the definitions and categories of the variables is provided in Appendix Table 2. | | | | | | |
